# A qualitative exploration of school-based staff’s experiences of delivering an alcohol screening and brief intervention in the high school setting: findings from the SIPS JR-HIGH trial

**DOI:** 10.1093/pubmed/fdy184

**Published:** 2018-10-29

**Authors:** G J McGeechan, E L Giles, S Scott, R McGovern, S Boniface, A Ramsay, H Sumnall, D Newbury-Birch, E Kaner, the SIPS JR-HIGH Study Team

**Affiliations:** 1 School of Social Sciences, Humanities and Law, Teesside University, Middlesbrough TS1 3BX, UK; 2 School of Health and Social Care, Teesside University, Middlesbrough TS1 3BX, UK; 3 Institute of Health and Society, Newcastle University, Newcastle NE2 4AX, UK; 4 Institute of Psychiatry, Psychology, & Neuroscience, King’s College London, London SE5 8AF, UK; 5 Faculty of Education, Health and Community, Liverpool John Moores University, Liverpool L3 2ET, UK

**Keywords:** adolescent alcohol consumption, alcohol screening and brief intervention, framework analysis, high-school intervention, normalization process theory, risky drinking

## Abstract

**Background:**

Whilst underage drinking in the UK has been declining in recent years, prevalence is still higher than in most other Western European countries. Therefore, it is important to deliver effective interventions to reduce risk of harm.

**Methods:**

Semi-structured interviews with staff delivering an alcohol screening and brief intervention in the high-school setting. The analysis was informed by normalization process theory (NPT), interviews were open coded and then a framework applied based on the four components of NPT.

**Results:**

Five major themes emerged from the analysis. The majority of participants felt that the intervention could be useful, and that learning mentors were ideally suited to deliver it. However, there was a feeling that the intervention should have been targeted at young people who drink the most.

**Conclusions:**

The intervention was generally well received in schools and seen as an effective tool for engaging young people in a discussion around alcohol. However, in the future schools need to consider the level of staffing in place to deliver the intervention. Furthermore, the intervention could focus more on the long-term risks of initiating alcohol consumption at a young age.

## Introduction

Whilst evidence suggests that the number of school aged children in the United Kingdom (UK) (11–18 years old), who drink alcohol has been declining in recent years,^1^ the prevalence of young people who drink alcohol in the UK remains amongst the highest in Europe.^2^ Furthermore, whilst overall prevalence is declining, those young people who do drink alcohol are drinking greater amounts than previously found.^3^

Underage drinking of alcohol has a number of negative consequences not only for underage drinkers themselves, but also for their families, and society as a whole.^4^ Initiating alcohol consumption before the age of 15 is associated with a number of negative outcomes such as poor quality of life, the development of alcohol use disorders, youth offending and risky sexual behaviour.^5–9^ Additionally, evidence suggests that the lower the age that a young person has their first drink of alcohol the more likely they are to develop alcohol related problems in later years.^10^

The high-school is seen as an appropriate setting to deliver interventions on substance use as they provide a captive audience who are used to receiving health and social care education.^11^ Interventions which are targeted at young people who are already drinking alcohol can be an effective and efficient strategy to reduce alcohol consumption, as the intervention is likely to be salient to those receiving it, more likely as they are, to be experiencing harm from alcohol consumption.^12–14^

Whilst previous research has examined the effectiveness of alcohol screening and brief interventions with young people,^15–17^ few studies have evaluated such interventions to understand the mechanisms which can lead to successful implementation, by exploring the experiences of school-based staff who deliver them.^18^ One such study highlighted that successful implementation of a school-based intervention depends on positive outcomes for at risk groups and does not necessarily require universal impact. Furthermore, the authors found that ease of delivery and user friendliness were essential components for securing engagement from school staff.^19^

Recently, researchers have been utilizing a sociological theory, normalization process theory (NPT) to evaluate the likelihood of new interventions becoming embedded into practice. This theory focusses on evaluating factors which facilitate and deter the implementation of new services, or interventions, into routine practice.^20^ Whilst primarily devised for use in clinical settings, it is increasingly being used in other settings such as social care.^21,22^ There are four core constructs within NPT which account for how people make sense of and comprehend new practice (coherence); how they implement and carry out the new practice (cognitive participation), the work that both individuals and wider organizations have to carry out to initiate the new practice (collective action), and their appraisals and reflections of it (reflexive monitoring).^23^

The present study was part of a larger, multi-site randomized controlled trial looking at the efficacy and effectiveness of school-based staff delivering alcohol screening and brief interventions to young people in high-schools across four regions of England (North East, North West, Kent and London).^24^

The primary aim of this study was to understand the mechanisms and processes of implementing an alcohol screening and brief intervention in the high-school setting. A qualitative evaluation, drawing on NPT, was conducted to explore staff perceptions of how the intervention could become embedded into the future work role of staff.

## Methods

Semi-structured interviews were conducted to explore school-based staff’s perceptions of conducting alcohol screening and brief interventions with young people. Interviews were conducted with staff involved in the trial: learning mentors (LMs) who delivered the control and intervention conditions to young people, and teaching staff who approved the study within their school.

### Intervention

The SIPS Jr-HIGH intervention is described in detail elsewhere.^24^ Briefly, LMs employed by schools were trained to deliver an alcohol screening and brief intervention, or control condition to young people aged 14–15 within the high-school setting. Participants completed an alcohol screening questionnaire, and those who scored positive for risky drinking were randomized into the trial. The intervention used motivational interview techniques to engage young people in discussions around alcohol use and to facilitate ‘change talk’ aimed at reducing alcohol consumption.

### Ethical approval

This study was approved by Teesside University’s School of Health and Social Care Research Governance and Ethics Committee in March 2016 (047/16).

### Recruitment

All LMs who delivered the intervention or control condition to young people in their school (*N* = 80), and all teaching staff who facilitated the trial (*N* = 30), were invited to take part in an interview. Invitation letters and information sheets were e-mailed to all eligible participants who were asked to complete a pro-forma indicating their age, ethnicity, job role and length of time in their current role. A sampling framework was created based on the pro-forma and we aimed to recruit a purposive sample of 24 participants: 12 LMs and 12 teachers, however, recruitment continued until data saturation had occurred.

### Data collection

A semi-structured interview topic guide was developed drawing on the four constructs of NPT: coherence, cognitive participation, collective action and reflexive monitoring.^23^ Additional questions were added to gain feedback on individual participants’ experiences of the trial. Interviews were conducted on school premises between June and July 2016 by one of four regional research co-ordinators.

### Analysis

Interviews were audio recorded and transcribed verbatim, with identifiable information removed from transcripts. Following transcription, interviews were analysed using framework analysis. Interviews were independently open coded by three researchers (G.M., S.B., A.R.) using QSR Nvivo version 11. These codes were then mapped onto a framework based on the four key constructs of NPT theory^23^ coherence, cognitive participation, collective action and reflective monitoring.

In order to ensure validity of the results, a proportion of the transcripts were second coded by an independent researcher. Any disagreement between first and second coders was resolved through discussion until consensus was met. Following second coding, the framework was further refined before the final themes were agreed by the research team.

## Results

### Participants

A total of 29 interviews were conducted. One interview included two LMs, due to time constraints within the school, therefore, the total number of participants was 30; 21 LMs (66.6% women); and 9 teachers (55.5% women). Most participants were of a white ethnic background (93.3%), and had been in their current job role for <5 years (43.3%). Interviews lasted on average 39 min (range = 12–102 min). Participant characteristics for the LM and teacher interviews can be seen in Table 1.

**Table 1:** Participant characteristics

Code	Gender	Ethnicity	Years in job	Site
Learning mentors (LM)				
LM1	Female	White	5–10	North West
LM2	Female	Black	>10	London
LM3	Female	White	<5	North West
LM4	Male	White	<5	North East
LM5	Male	White	5–10	North East
LM6	Female	White	>10	North West
LM7	Female	White	>10	Kent
LM8	Male	White	<5	North East
LM9	Female	White	<5	Kent
LM10	Female	White	<5	Kent
LM11	Female	White	>10	North East
LM12	Female	White	5–10	North West
LM13	Male	White	>10	North East
LM14	Female	White	>10	North West
LM15	Female	White	>10	North West
LM16	Male	White	>10	North East
LM17	Male	White	<5	Kent
LM18	Female	White	<5	Kent
LM19	Male	White	<5	North East
LM20	Female	White	<5	Kent
LM21	Female	White	5–10	Kent
Teachers (T)				
T1	Male	White	<5	London
T2	Female	White	5–10	Kent
T3	Female	White	<5	London
T4	Male	White	5–10	North West
T5	Male	White	<5	North East
T6	Female	White	5–10	Kent
T7	Male	Mixed	<5	North West
T8	Female	White	5–10	North East
T9	Female	White	>10	North West

### Interviews

**Figure 1:**
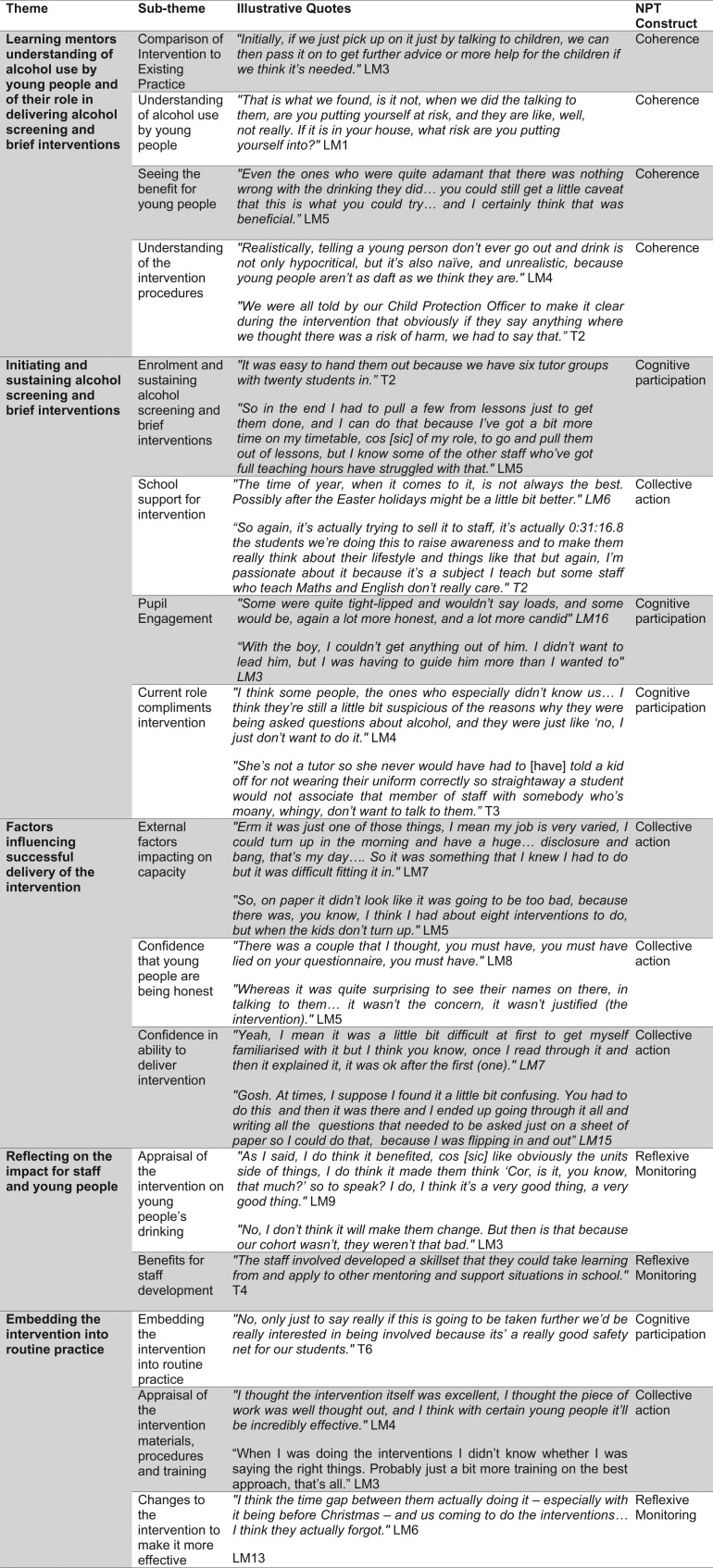
Illustrative quotations.

In total we identified five themes, each with a number of sub-themes which are explained in more detail below. Table 2 highlights how these themes map onto the core constructs of NPT,^23^ whilst Fig. 1 provides illustrative quotes.

**Table 2:** Mapping of themes from LM and teacher interviews to core constructs of NPT

Theme	Sub-theme	NPT constructs
LM understanding of alcohol use and their role in delivering alcohol screening and brief interventions	Comparison of intervention to existing practice	Coherence
	Understanding of alcohol use by young people	
	Seeing the benefit for young people	
	Understanding of the intervention procedures	
Initiating and sustaining alcohol screening and brief interventions	Enrolment and sustaining alcohol screening and brief interventions	Cognitive participation
	Pupil engagement	
	School support for intervention	Collective action
	Current role compliments intervention	Cognitive participation
Reflecting on the impact for staff and young people	Appraisal of the intervention on young people’s drinking	Reflexive monitoring
	Benefits for staff development	
Factors influencing successful delivery of intervention	External factors impacting on capacity	Collective action
	Confidence that young people are being honest	
	Confidence in ability to deliver intervention	
Embedding intervention into routine practice	Embedding intervention into routine practice	Cognitive participation
	Changes to intervention to make it more effective	Reflexive monitoring
	Appraisal of the intervention materials, procedures and training	Collective action

## LMs understanding of alcohol use by young people and of their role in delivering alcohol screening and brief interventions

### Comparison of intervention to existing practice (coherence)

There was a level of variation in the roles undertaken by LMs, ranging from purely pastoral roles, to those with teaching responsibilities. This resulted in differing levels of experience in discussing alcohol use with young people. However, in general LMs only addressed such issues when linked to a specific incident. Therefore, whilst teaching staff felt that LMs had appropriate skills to facilitate the interventions, one-to-one interventions for alcohol use were not common practice.

### Understanding of alcohol use by young people (coherence)

There was shared a belief amongst LMs that young people do not drink alcohol to the same extent as previous generations. This would likely impact on their views of the benefits of alcohol specific interventions if they did not believe young people were drinking to the extent where interventions were necessary. There was also a tendency to focus on immediate short-term risks associated with young people’s drinking suggesting that LMs possessed a narrow interpretation of the aims of the intervention.

### Seeing the benefit for young people (coherence)

However, despite feelings that alcohol use amongst young people had decreased, and a lack of concern for those who drink in a ‘safe’ environment, there was still a belief that it was important to provide education on the risks associated with drinking alcohol. This would ensure they were equipped with appropriate knowledge and skillsets to make informed decisions about alcohol in the future.

### Understanding of intervention procedures (coherence)

Most participants demonstrated an awareness of their role in delivering the alcohol screening and brief intervention, and the control condition. For LMs there was an understanding that telling young people not to drink would have limited impact: ‘We want it to be a dialogue… there is absolutely no point in just going, don’t have sex until you are 16, don’t drink until you are 18.’

Instead, LMs understood the need to engage young people in open and honest discussions to raise their self-efficacy in relation to drinking. For teaching staff there was an acceptance of the need for confidentiality and trust between the LMs and young people.

## Initiating and sustaining alcohol screening and brief interventions

### Enrolment and sustaining alcohol screening and brief interventions (cognitive participation)

Initiation of the overall trial within schools was perceived as straightforward by LMs and teachers. The initial screening survey was easy to arrange and required no more than someone handing out the surveys to pupils: ‘nobody had more than one class at a time to put through the questionnaire, I don’t think it was dreadfully difficult to organize and do’. However, the initiation and maintenance of the intervention and control conditions was more complicated, and varied between schools. One factor which affected this was the availability of appropriately trained staff. For example, one school had two LMs responsible for arranging 42 appointments with young people, whilst another had four LMs but only nine appointments.

### School support for intervention (collective action)

Support from teaching staff within the school was key for initiating and sustaining the brief intervention and control conditions. However, not all teaching staff had been made aware that the trial was being conducted. Furthermore, the interventions and follow-up surveys coincided with school exams which could have been a barrier when arranging for young people to attend their appointment. However, as one LM described, this was not always an issue: ‘Teachers are really good here; unless they’ve really got something, they’re happy to work with you. No, I didn’t have any trouble at all.’

### Pupil engagement (cognitive participation)

Most participants felt that young people were willing to engage in the intervention sessions, with few discussing occasions when someone had refused participation. However, there were differences in levels of engagement of young people, with some happy sharing their thoughts and feelings on alcohol use, whilst others were more reserved: ‘Some were quite tight-lipped and wouldn’t say loads, and some would be, again a lot more honest, and a lot more candid.’

### Current role compliments intervention (cognitive participation)

Participants discussed the legitimacy of LMs undertaking this work given their pastoral role within the school. Whilst they have a less formal relationship with some students, they demonstrated an awareness that some pupils still view them as staff and would perhaps respond better to an outside agency. Teaching staff were more confident, however, that the pastoral relationship between the LMs and pupils would be of benefit when delivering the intervention.

## Factors influencing successful delivery of the intervention

### External factors impacting on capacity (collective action)

Even when the trial had begun, and intervention and control sessions were arranged, an incident could occur in school requiring LMs involvement as a member of the pastoral team. Furthermore, young people forgot to turn up for sessions, which meant extra time spent rearranging missed appointments. Some schools also discussed financial difficulties impacting on how many pastoral staff were employed, impacting on the capacity of remaining staff: ‘They are an academy, and it’s proving to be quite difficult financially, there’s been a lot of cost cuts, a lot of staff gone.’

### Confidence that young people were being honest (collective action)

LMs discussed concerns they had over the recruitment process for the intervention suggesting that many young people did not report drinking a lot of alcohol, therefore did not warrant an intervention. Most LMs accepted that the young people were being honest with few considering that they may have been reluctant to disclose the full extent of their drinking to them: ‘I’ve no reason to believe these young people were lying… Mostly it was young people saying “Well, I don’t really drink a lot”, to be honest’. Instead, most felt that either the screening tool was too sensitive, identifying people with low levels of alcohol consumption, or the young person had simply lied on the screening tool.

### Confidence in ability to deliver the intervention (collective action)

The interviews with LMs highlighted that they grew in confidence as they progressed through the intervention and control sessions with young people. There was a lot of paperwork which had to be filled out for the sessions, and this could sometimes be confusing. One participant explained that it was often difficult to remember the exact process: ‘Yeah, I mean it was a little bit difficult at first to get myself familiarized with it but I think you know, once I read through it and then it explained it, it was ok after the first [one]’.

## Reflecting on the impact for staff and young people

### Appraisal of the intervention on young people’s drinking (reflexive monitoring)

When asked to evaluate the potential impact of the intervention on young people’s drinking there were mixed feelings expressed by LMs. Some felt that it could potentially be beneficial for students and that it was a useful tool for engaging young people in discussions around alcohol. As one participant put it: ‘I actually think they might think about that drink before they have it from now on’. However, LMs were not sure whether the intervention would have any lasting impact.

### Benefits for staff development (reflexive monitoring)

An interesting theme to emerge from the interviews centred on a recognition amongst teaching staff that participating in the trial could provide benefits for the LMs. It was seen as an opportunity for them to develop new skills which could be used in other aspects of their job: ‘I think furthermore it’s also meant that our LMs to perhaps more quickly, more promptly look at some of the macro-issues around that child’.

## Embedding the intervention into routine practice

### Embedding the intervention into routine practice (cognitive participation)

A number of participants would have been happy to support future implementation of the intervention, however, there were a number of factors which would need to be considered. Schools would need to factor in the number of staff who could be trained to deliver the intervention. ‘The staffing that we have in place, around the number of students that we have would need to be looked at.’

### Appraisal of the intervention materials, procedures and training (collective action)

The training provided to LMs before delivering the intervention and control conditions was also generally well received. There was an acknowledgement that the training, and study materials were well thought-out, and left LMs prepared for their appointments with young people. However, the timing of the training could be adapted in the future as in some schools this took place several months before any interventions which may have resulted in a loss of confidence.

### Changes to the intervention to make it more effective (reflexive monitoring)

Whilst the feedback from LMs and teachers on the intervention materials was generally positive, there were some suggestions of changes which could be made to make the intervention more effective in the future. For example, the length of time it took to progress from the baseline screening survey to meeting one-to-one with young people in delivering the intervention and control sessions. Whilst the survey generally took place before Christmas, the intervention and control appointments were not arranged until around Easter. Furthermore, some LMs felt that the school should be able to target the intervention to those whom they feel would benefit, rather than screening all of their students for alcohol use: ‘But I certainly think that everyone in the school should be made aware of stuff and then targeted, more targeted intervention with people’.

## Discussion

### Main findings of the study

In this study we drew on the four constructs of NPT to evaluate the delivery of an alcohol screening and brief intervention in the high-school setting;^23^ (coherence, cognitive participation, collective action, reflexive monitoring). In general, LMs were identified as ideally situated to deliver the SIPS Jr-High intervention. High coherence was displayed by LMs who clearly understood the differences between their current role and the new intervention they were tasked with delivering. Teaching staff felt that LMs had a particular skillset, and unique relationship with young people which allowed them to deliver the intervention.

There was consensus that most young people receiving the intervention were not drinking alcohol at levels LMs would consider merited such an intervention. This could impact on their engagement with the intervention if they did not feel it was warranted (cognitive participation). They instead reflected that targeting the intervention at students whom they felt would benefit would be a more effective strategy (reflexive monitoring).

### What is already known on this topic?

Alcohol consumption amongst young people is declining, however, those who drink alcohol are drinking in ever increasing quantities meaning effective interventions are still necessary.^3^

Evidence suggests that practitioners must see the benefit of a new intervention or they can become resistant to implementation.^25^ Therefore, it is essential that delivery staff are aware of the short and long-term risks of alcohol consumption in this age group in order to see the benefit of such interventions.

### What does this study add?

This is the first study to our knowledge which has adopted a theory based evaluation of the processes and mechanisms likely to impact on embedding an alcohol screening and brief intervention in the high school setting. By exploring staff experiences we have been able to highlight the need for schools to consider staffing resources before implementing a targeted intervention, and also the need to build the intervention around existing time-tables to avoid clashes with end of year exams. Most importantly, we highlighted that LMs have the appropriate skillset, and relationship with students to deliver such an intervention. This is important for policy and practice partners who may consider commissioning external alcohol services for schools by highlighting that staff within the school setting already possess the skills required to deliver interventions. Future research could explore the impact of school delivered alcohol interventions out with a randomised controlled trial setting.

### Limitations

Whilst we aimed to recruit as wide a sample as possible in terms of gender, ethnicity, job role and experience our sample was predominately White British. Furthermore, recruitment from one study site in particular proved more challenging, however this was reflective of the trial as a whole.

Furthermore, NPT was initially developed in relation to health-care contexts and its use in the high-school setting is limited.^26^ Other implementation models have been posited such as implementation climate,^27^ absorptive capacity^28^ and organizational readiness,^29^ which may also have been relevant theoretical constructs for this research. However, it was felt that NPT’s particular focus on healthcare would be most relevant to a study looking at delivering an alcohol screening and brief intervention.
